# Psychosocial, Mental, and Suboptimal Health Measures Are Not Affected by Provision of Abdominal Aortic Calcification Imaging Results: A 12-Week RCT

**DOI:** 10.1016/j.focus.2026.100507

**Published:** 2026-05-09

**Authors:** Simone Radavelli-Bagatini, Lauren C. Blekkenhorst, Catherine P. Bondonno, Jack Dalla Via, Marc Sim, Abadi K. Gebre, James Dimmock, John T. Schousboe, Richard J. Woodman, Kun Zhu, Pawel Szulc, Ben Jackson, Wei Wang, Markus P. Schlaich, Douglas P. Kiel, Wai H. Lim, Amanda Devine, Moira Sim, Robin M. Daly, Jonathan M. Hodgson, Joshua R. Lewis

**Affiliations:** 1 Nutrition and Health Innovation Research Institute, School of Medical and Health Sciences, Edith Cowan University, Joondalup, Australia; 2 Medical School, The University of Western Australia, Perth, Australia; 3 Institute for Physical Activity and Nutrition (IPAN), School of Exercise and Nutrition Sciences, Deakin University, Geelong, Australia; 4 Department of Psychology, College of Healthcare Sciences, James Cook University, Townsville, Australia; 5 Park Nicollet Clinic and HealthPartners Institute, Minneapolis, Minnesota; 6 Division of Health Policy and Management, University of Minnesota, Minneapolis, Minnesota; 7 Flinders Centre for Epidemiology and Biostatistics, Flinders University, Adelaide, Australia; 8 Department of Endocrinology and Diabetes, Sir Charles Gairdner Hospital, Nedlands, Australia; 9 INSERM UMR 1033, University of Lyon, Hospices Civils de Lyon, Lyon, France; 10 The Kids Research Institute Australia, Perth, Australia; 11 School of Human Sciences (Exercise and Sport Science), University of Western Australia, Perth, Australia; 12 School of Medical and Health Sciences, Edith Cowan University, Joondalup, Australia; 13 Dobney Hypertension Centre, Medical School, Royal Perth Hospital Research Foundation, Perth, Australia; 14 Department of Nephrology, Royal Perth Hospital, Perth, Australia; 15 Department of Cardiology, Royal Perth Hospital, Perth, Australia; 16 Hinda and Arthur Marcus Institute for Aging Research, Hebrew SeniorLife, Boston, Massachusetts; 17 Department of Medicine, Beth Israel Deaconess Medical Center, Harvard Medical School, Boston, Massachusetts; 18 Department of Renal Medicine, Sir Charles Gairdner Hospital, Perth, Australia

**Keywords:** Vascular calcification, provision of disease risk, mental health, suboptimal health status, well-being, psychosocial health

## Abstract

**Introduction:**

The provision of abdominal aortic calcification imaging results can elicit cardiovascular disease risk–reducing behaviors. However, its impact on psychosocial, mental, and suboptimal health measures is less clear.

**Methods:**

Secondary analyses of a previously reported 12-week, single-blind RCT including 240 community-dwelling older Australian males and females were conducted. Participants were provided with abdominal aortic calcification imaging results and cardiovascular disease risk–reducing educational resources (abdominal aortic calcification+education, *n*=121) or education alone (control+education, *n*=119). Psychosocial distress, mental health score, and suboptimal health status were estimated using validated questionnaires. Linear mixed models were used to compare changes between groups across 12 weeks.

**Results:**

A total of 240 participants (mean±SD age=67.8±5.0 years; 57.5% female) were randomized, and 227 (95%) completed the study. Approximately 57% had evidence of abdominal aortic calcification (≥1 on a 24-point scale) at baseline. Providing abdominal aortic calcification imaging results to older males and females did not affect psychosocial health, mental health, and suboptimal health measures compared with control+education (all *p*>0.05). The mean net differences for change (95% CI) for depression, anxiety, and stress were −0.50 (−1.42, 0.42), −0.20 (−0.81, 0.41), and −0.21 (−1.18, 0.76), respectively; the mean net difference for change (95% CI) for mental health component score was 1.69 (−1.29, 4.67), and for total suboptimal health status, it was −0.84 (−2.08, 0.39).

**Conclusions:**

In older males and females, the provision of abdominal aortic calcification, along with education on cardiovascular disease risk factors, did not alter measures of psychosocial, mental, and suboptimal health status over 12 weeks compared with the provision of education alone. The study was registered at www.anzctr.org.au ACTRN12618001087246.

## HIGHLIGHTS

• Vascular disease assessment can be incorporated into routine bone density testing.
• In Australia, over 700,000 people undergo bone density testing annually.
• Providing vascular disease imaging results did not alter mental well-being measures.
• Vascular disease imaging results may be used to elicit healthy lifestyle changes.
• Behavior change programs should provide both disease risk and lifestyle education.

## Graphical Abstract

**Figure:**
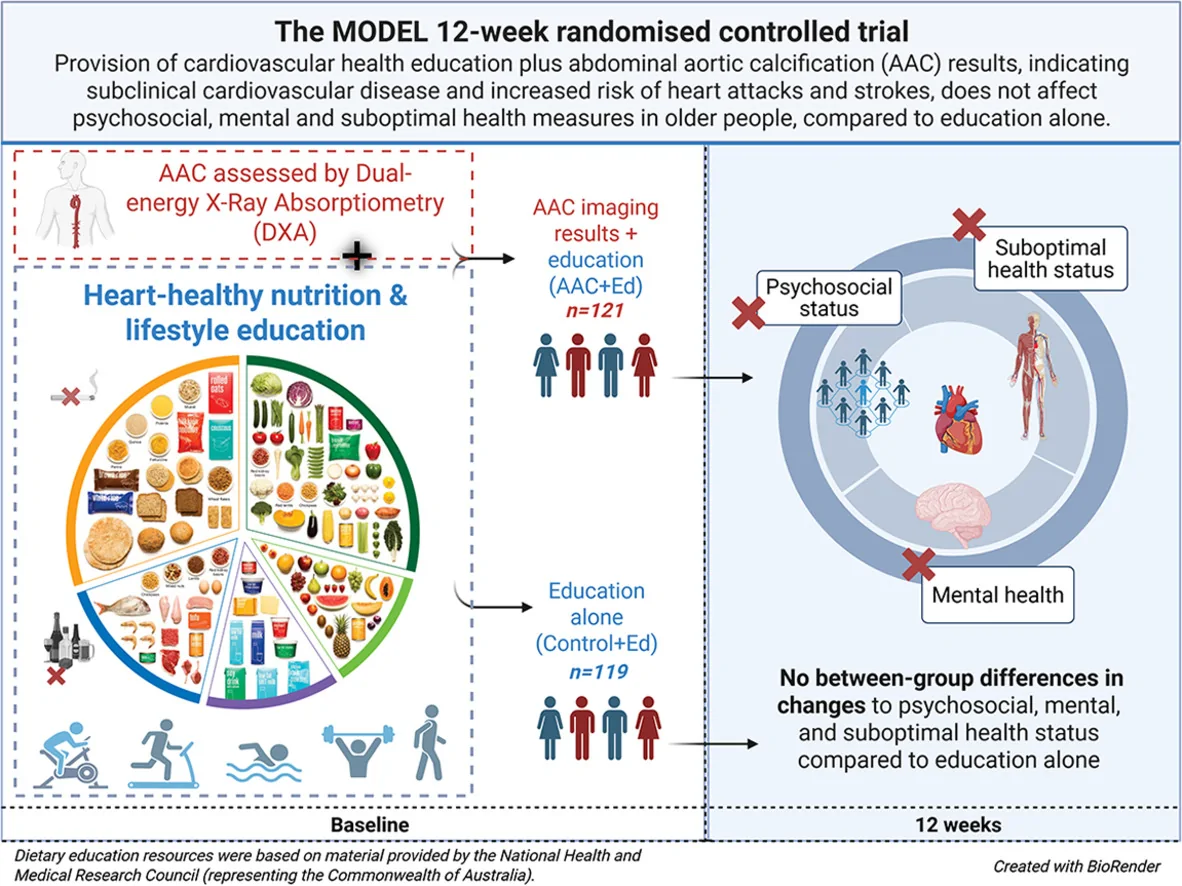

## INTRODUCTION

Despite widespread knowledge that a healthy diet and lifestyle^1^ can reduce the risk of chronic diseases such as cardiovascular disease (CVD),^2^ most people do not successfully achieve and sustain a healthy lifestyle, especially with regard to diet and physical activity.^3^ This is commonly observed in behavior change interventions,^4^ where participants often fail to adhere to guidelines, resulting in a lack of meaningful changes.^5^ For CVD prevention, new and efficient strategies for both short- and long-term changes in diet and lifestyle behaviors are needed.

Awareness of CVD risk may encourage individuals to make positive changes in lifestyle behaviors, improving health outcomes.^6^ For example, provision of carotid atherosclerosis and coronary artery calcification imaging results has been shown to improve blood pressure control and circulating lipid levels as well as medication initiation and adherence.^7^, ^8^, ^9^, ^10^, ^11^, ^12^

In the Modification Of Diet, Exercise and Lifestyle (MODEL) study, significant improvements in lipid levels and CVD risk score were observed over 12 weeks after the provision of visual information and explanation of abdominal aortic calcification (AAC) results, as previously reported.^13^ AAC is a measure of structural vascular disease,^14^ characterized by the deposition of calcium in the abdominal aorta, which is one of the first sites where vascular calcification is seen.^15^ AAC is a measure of subclinical CVD^16^^,^^17^ and predicts future CVD events and all-cause mortality,^18^ affecting >30% of people aged 45–54 years and up to 90% of people aged >75 years.^16^ A meta-analysis reported AAC prevalence to be higher in older people with underlying conditions such as Type 2 diabetes (84%) or chronic kidney disease (97%).^18^

Provision of vascular imaging results can increase the awareness of an individual’s risk of having a heart attack or stroke and assist in planning for and making decisions to prevent future clinical events.^19^^,^^20^ However, providing health information to individuals, particularly results that indicate susceptibility to health issues, can be perceived as a threat.^21^ Although some individuals do respond positively when exposed to a threat and are motivated to act, others respond negatively and are more vulnerable to psychological harm, worry, and distress, leading to a reduced quality of life.^21^^,^^22^ Pooled data from a meta-analysis including 127 articles (*n*=27,372) demonstrated that fear-inducing content aiming to influence attitudes, intentions, and behaviors is unlikely to lead to undesirable outcomes.^23^ In terms of the provision of vascular disease information, some studies report no changes in psychosocial distress and quality of life,^24^, ^25^, ^26^ whereas others report increased worry and psychological distress.^9^^,^^27^ Given the conflicting (and limited) evidence available,^28^ this study aims to determine the impact of the provision of AAC imaging results with education on CVD risk factors on changes in psychosocial, mental, and suboptimal health measures over 12 weeks compared with that of education alone in older Australian males and females from the MODEL study. This is important to inform evidence-based risk communication strategies and to help clarify the potential mechanism(s) driving any changes in dietary and lifestyle behaviors related to CVD.

## METHODS

### Study Sample

As previously reported,^13^ the MODEL study was a 12-week, single-blind (researchers who were involved in data collection were blinded to treatment allocation, whereas the researcher who assigned the codes and the participants themselves were not blinded) RCT. The study aimed to compare the impact of providing information on AAC imaging results plus educational resources (i.e., AAC+Ed) on CVD risk factors with that of education alone (i.e., Control+Ed) on changes in fruit and vegetable measures (primary outcomes) and other CVD risk-reducing behaviors (i.e., other aspects of the diet, physical activity, blood pressure, lipid levels, and others). A total of 240 community-dwelling Australian males and females aged 60–80 years were recruited for the study, and 227 (95%) completed the 12-week RCT. The methodology of the study has been previously reported.^10^^,^^13^ The study flow chart is shown in Appendix Figure 1 (available online). This study reports findings from planned secondary analyses investigating the impact of the provision of AAC imaging results on psychosocial, mental, and suboptimal health measures over 12 weeks, compared with that of the provision of education alone. Although the analyses were preplanned,^10^ they were not included among the secondary outcomes highly related to the primary outcomes previously reported,^13^ because the topic fell outside the scope of that previously published paper^13^ owing to addressing a different research question. This study was conducted during the coronavirus disease 2019 (COVID-19) pandemic, which may have affected the results of the study, as previously reported.^13^ The study was registered with the Australian New Zealand Clinical Trial Registry (ACTRN12618001087246) and received approval from the Edith Cowan University Human Research Ethics Committee (Project Number 20513 HODGSON) in August 2020. The CONSORT statement^29^ and the critical appraisal tool for the assessment of risk of bias for RCTs^30^ were used to report the results of this trial. The CONSERVE checklist for reporting results of clinical trials that underwent substantial changes owing to extenuating events, such as the COVID-19 pandemic, has been previously reported.^13^

Community-dwelling males and females residing in Western Australia were recruited through newspaper advertisements and existing participant databases. Eligibility of participants was checked through a phone call; participants were considered eligible if they were aged 60–80 years, had a mobile phone and access to an email address, and were willing to attend clinic visits and complete online surveys. Consent for participation was provided by all participants at the first clinic visit at Sir Charles Gairdner Hospital Bone Density Unit (Perth, Australia) before starting the baseline assessments. Three recruitment intakes were conducted: Wave 1 (September 2020 to April 2021), Wave 2 (May 2021 to September 2021), and Wave 3 (October 2021 to May 2022), with each wave comprising 75–90 participants.

### Intervention

Participants were randomly allocated (1:1) to either the AAC+Ed (received information on AAC imaging results plus educational resources) or Control+Ed (education alone) at baseline. A statistician (RJW) generated the allocation sequence using random block sizes of either 4 or 6 and with the aid of the user-written module ralloc in Stata (StataCorp).^31^ Sequential treatment codes were assigned to participants enrolling individually or to those in the same block if enrolling with a partner (e.g., spouses or siblings). At baseline, a research assistant who was blinded to the intervention enrolled participants and performed the clinical assessments, whereas an unblinded investigator, who was not involved in data collection, assigned the numeric codes. Participants were not blinded to treatment.

Approximately 2 weeks after the baseline clinic visit, participants received a prescheduled email informing them of their group allocation. For both groups, the email also contained a copy of a dietary and lifestyle booklet, a link to a ∼12-minute video, and a document with frequently asked questions. These educational resources (booklet and video) provided to both groups comprised information on CVD^32^ plus dietary and physical activity^33^ recommendations based on the Australian Dietary Guidelines^34^ and the Heart Foundation.^32^ The AAC+Ed group had a slightly longer video that included information about AAC. For the AAC+Ed group only, a letter with participants’ AAC imaging results was enclosed in the email, along with an explanatory letter to their general practitioner (these were also emailed directly to participants’ doctors). Participants in the Control+Ed group received their AAC imaging results at the end of the 12-week study.

An example of the AAC imaging results letter has been previously published.^13^ Three different letters were provided to participants: (1) no evidence of AAC (AAC=0), displaying a green circle and a copy of the lateral spine image where the abdominal aorta could not be seen next to the spine, owing to the absence of calcification; (2) evidence of AAC (AAC 1–5), presenting a red circle, arrows pointing to the areas where calcification was seen, plus a diagram representing the calcified areas; and (3) evidence of extensive AAC (AAC ≥6), which was similar to 2 but displayed a larger area of calcification. The results and/or information were sent to participants through email in the morning, followed by a scheduled afternoon phone call to discuss the information, on the same day that the participants were sent the email.

### Measures

Participants' demographics were assessed using online questionnaires as previously reported.^13^ At the baseline and 12-week clinic visits, Dual-energy X-ray Absorptiometry scans were performed at Gairdner Bone Densitometry Services, Sir Charles Gairdner Hospital (Perth, Australia) using a Hologic Horizon A densitometer (Hologic, Inc., Bedford, MA). A lateral spine image was used to assess AAC, and the images were read and scored by an expert in densitometric imaging (JTS). AAC was semiquantitatively assessed using the Kauppila scoring system (ranging from 0–24; AAC24),^35^ which was used to determine whether participants had no evidence of AAC (AAC=0), low-moderate AAC (AAC=1–5), or evidence of extensive AAC (AAC≥6).

All outcomes in this study were self-reported by participants. Psychosocial, mental, and suboptimal health measures were assessed using online questionnaires (through the Qualtrics platform) at baseline and 12 weeks, which participants completed at home at their own pace. All participants completed their baseline questionnaires prior to being randomized (i.e., prior to receiving AAC results for the AAC+Ed group). These measures were derived from 3 different questionnaires validated for the Australian population. The Depression Anxiety Stress Scales (DASS)21 is a 21-item self-report questionnaire designed to measure symptoms of depression, anxiety, and stress^36^ (not intended for clinical diagnoses) in the preceding week. Each of the 3 DASS-21 scales contains 7 items. The total DASS-21 score was calculated by summing the scores for the relevant items multiplied by 2 (with a maximum score of 42 in each domain, ranging from to 0 to 126, in accordance with the original DASS-42).^36^ The cut off scores indicate mild, moderate, severe, and extremely severe scores for each DASS scale (Appendix Table 1, available online).^36^ Values ≥28, ≥20, and ≥34 indicate an elevated risk of depression, anxiety, and stress scores, respectively.^36^

The 36-item Short Form Health Survey (SF-36)^37^ forms 8 different domains, which can be used to estimate 2 components: a physical component score (including 4 domains: functioning, role‐physical, bodily pain, general health) and a mental health component summary (including 4 domains: vitality, social functioning, role‐emotional, and mental health).^38^ Only the mental health component scores of the SF-36 were reported for this study.^38^ The total mental health component summary score ranges from 0 to 100 (poor to best health), with a score <46 indicating poorer mental health and ≥47 indicating good mental health for the general Australian population.^38^^,^^39^

The Suboptimal Health Status Questionnaire-25 (SHSQ-25)^40^ provides a measure of overall health. Suboptimal health status is a physical stage between health and disease characterized by perceived health complaints that occur (and may be reversible) before the onset of chronic diseases.^41^ It is composed of 25 items and includes 5 domains: fatigue (9 items), cardiovascular system (3 items), digestive tract (3 items), immune system (3 items), and mental status (7 items). Participants rated their health on a 5-point Likert scale on the basis of how frequently those issues occurred in the previous 3 months (Appendix Table 2, available online). Response options for each statement included 0, never or almost never; 1, occasionally; 2, often; 3, very often; and 4, always. A total score ranging from 0 to 100 (good to poor suboptimal health) was estimated by summing the scores from the 25 items. Scores for each specific domain were estimated by summing the scores for the subscale items. The median has been used as a cut off and indicates low versus high total suboptimal health scores.^42^

Sleep quality was assessed using the Pittsburgh Sleep Quality Index, a questionnaire used to measure the quality and patterns of sleep in older adults.^43^ Participants scored each question on a 4-point Likert scale for the previous month, from 0, no difficulty, to 3, severe difficulty. A global score was estimated by summing all items from the questionnaire (ranging from 0 to 21, with higher scores indicating worse sleep quality).

### Statistical Analysis

Data analyses involved an intention-to-treat method including all participants randomized, using Stata statistical software (Version 18.0; Stata, College Station, TX). Descriptive statistics were used to summarize baseline and 12-week values. The effect of the intervention on the outcomes was analyzed using a linear mixed-effects model. The models included fixed effects for group (AAC+Ed and Control+Ed) and time (baseline and 12 weeks) and their interaction. Postestimation margins, after the mixed-effects regression models, were used to determine within-group changes over 12 weeks for each outcome variable, and the effect of the intervention was determined using the results for the treatment group × time interaction term. The mean net difference between groups and the respective *p*-values were estimated using linear mixed models, with values representing means and 95%CI.

For additional sensitivity analyses, the Spearman Rho (ρ) test was used to further investigate the associations between higher AAC scores (continuous) at baseline and changes over 12 weeks in outcome variables (continuous) within the AAC+Ed group. To determine whether the presence of AAC in the AAC+Ed group had any influence on outcome measures, Mann–Whitney *U* tests were used to examine the differences between changes over 12 weeks for each of the outcomes by presence/absence of AAC (AAC=0 vs AAC≥1). The normality of the distribution of continuous variables was assessed using skewness and kurtosis. Values of skewness and kurtosis were checked, and an overall *p*<0.05 was interpreted as indicative of approximate non-normality. In addition, visual inspection of histograms and Q–Q plots was conducted to support these tests. Statistical significance was set at *p*<0.05 for all analyses.

## RESULTS

The results for the primary outcomes (change in fruit and vegetable measures) and secondary outcomes related to the primary outcomes (other CVD risk-reducing behaviors such as physical activity, lipid levels, and blood pressure) of the MODEL study have been previously reported.^13^ This study presents secondary results of the MODEL RCT investigating whether provision of AAC imaging results (vascular disease risk) has any beneficial or harmful effects on measures of psychosocial, mental, and suboptimal health.

Baseline characteristics of this study have been reported previously^13^ and were similar in both groups. Additional characteristics are shown in Table 1. Briefly, the trial included 245 participants, of whom 240 were randomized to either the AAC+Ed (*n*=121, provided with AAC imaging results and educational resources) or the Control+Ed (*n*=119, provided with educational resources alone). A total of 227 (95%) completed the 12-week study. Dropout rates were similar in both the AAC+Ed and Control+Ed groups (5.0% vs 7.2%, respectively). Participants’ mean±SD age was 67.8±5.0 years, and 57.5% were female. Approximately 57% had evidence of AAC (scores >1), with <3% (*n*=6 of 240) presenting with evidence of extensive AAC (scores ≥6).

**Table 1:** Baseline Characteristics of Participants in the AAC+Ed and Control+Ed Groups

Characteristics	AAC+Ed *n*=121	Control+Ed *n*=119
Age (years), mean±SD	68.0±4.9	67.5±5.1
BMI (kg/m^2^), mean±SD	26.7±4.7	27.7±4.9
Gender (females), *n* (%)^a^	70 (57.8)	68 (57.1)
Relationship status, *n* (%)		
Married	79 (65.3)	79 (66.4)
Other^b^	42 (34.7)	40 (33.6)
Level of education, *n* (%)		
Never to some high school	7 (5.8)	7 (5.9)
Completed high school	19 (15.7)	21 (17.6)
Technical, diploma, or certificate	41 (33.9)	48 (40.3)
Bachelor’s degree	35 (28.9)	29 (24.4)
Higher degree	15 (12.4)	14 (11.8)
Not specified	4 (3.3)	0
Socioeconomic status, *n* (%)		
<$37,000	42 (34.7)	35 (29.4)
$37,000–$90,000	51 (42.2)	52 (43.7)
>$90,000	28 (23.1)	32 (26.9)
Smoking status, *n* (%)		
Never smoker	70 (57.9)	67 (56.3)
AAC, *n* (%)		
AAC=0	51 (42.1)	52 (43.7)
AAC≥1	70 (57.9)	67 (56.3)
DASS^c^		
Depression categories, *n* (%)		
Normal (scores 0–9)	102 (84.3)	104 (87.4)
Mild (scores 10–13)	9 (7.4)	7 (5.9)
Moderate (scores 14–20)	7 (5.8)	5 (4.2)
Severe (scores 21–27)	3 (2.5)	3 (2.5)
Extremely severe (score ≥28)	0	0
Depression scores, mean±SD		
Total	4.0 ± 5.4	3.9 ± 5.4
Normal	2.0 *±* 2.3	2.3 *±* 2.5
Mild	10.7 *±* 1.0	10.9 *±* 1.1
Moderate	15.1 *±* 1.1	16.0 *±* 2.8
Severe	24.7 *±* 1.2	25.3 *±*1.2
Anxiety categories, *n* (%)		
Normal (scores 0–7)	109 (90.1)	109 (91.6)
Mild (scores 8–9)	8 (6.6)	5 (4.2)
Moderate (scores 10–14)	3 (2.5)	4 (3.4)
Severe (scores 15–19)	1 (0.8)	1 (0.8)
Extremely severe (scores ≥20)	0	0
Anxiety scores, mean ± SD		
Total	2.5 *±* 3.1	2.4 *±* 3.0
Normal	1.8 ± 2.0	1.7 ± 1.8
Mild	8 ± 0	8 ± 0
Moderate	10 ± 0	11 ± 1.2
Severe	18 ± 0	16 ± 0
Stress categories, *n* (%)		
Normal (scores 0–14)	114 (94.2)	116 (97.5)
Mild (scores 15–18)	5 (4.1)	3 (2.5)
Moderate (scores 19–25)	2 (1.6)	0
Severe (scores 26–33)	0	0
Extremely severe (scores ≥34)	0	0
Stress scores, mean±SD		
Total	5.8 ± 5.3	4.9 ± 4.7
Normal	5.0 ± 4.3	4.6 ± 4.3
Mild	16.4 ± 0.9	17.3 ± 1.2
Moderate	23.0 ± 1.4	-
MCS categories, *n* (%)^d^		
Better mental health (scores ≥47)	111 (92.5)	111 (93.3)
Poorer mental health (scores <47)	9 (7.5)	8 (6.7)
MCS scores, mean±SD		
Total	80.4±16.7	81.5±16.5
Better mental health	83.8±12.2	84.8±11.2
Poorer mental health	38.6±3.8	35.8±6.3
SHS categories, *n* (%)^e^		
Poorer suboptimal health (scores ≥37)	66 (54.6)	60 (50.4)
Better suboptimal health (scores <37)	55 (45.5)	59 (49.6)
SHS scores, mean±SD		
Total	38.6±8.4	37.9±8.0
Poorer suboptimal health	44.4±6.8	44.1±6.4
Better suboptimal health	31.7±3.4	31.6±3.4
Sleep quality index, mean±SD	6.5±4.0	5.4±3.2

*Note*: Values are presented as *n* (%) or mean±SD. They were estimated using descriptive statistics (continuous variables) or chi-square test (categorical variables). AAC-24 scoring using bone density scans has been previously validated^14^ and extensively used.

a Gender refers to how a person identifies themself.

b Other includes single, divorced, widowed, or de facto.

c The DASS-21 score is calculated by summing the scores for the relevant items for each domain multiplied by 2 (maximum score of 42 in each domain), with values ≥28, ≥20, and ≥34 indicating an elevated risk of depression, anxiety, and stress scores, respectively.^36^

d Total MCS score ranges from 0 to 100 (poor to best mental health), with a cut off of 47 indicating poorer (<47) versus better (≥47) mental health for the general Australian population.^38^^,^^39^

e The total SHS score ranges from 0 to 100 (best to poor suboptimal health),^40^ with the median being used as a cut off and indicating better (<37) versus poorer (≥37) health status/well-being. AAC, abdominal aortic calcification; DASS, Depression, Anxiety, Stress Scale; Ed, education; SHS, suboptimal health status; MCS, mental health component summary.

There were no significant group differences for changes over 12 weeks in psychosocial, mental, and suboptimal health measures between groups (Table 2, Table 3, Table 4). However, small significant within-group changes over 12 weeks were observed in psychosocial measures assessed by the DASS-21, such as a decrease in depression and stress scores in the AAC+Ed group (mean [95% CI]= −1.00 [−1.65, −0.36] and −0.69 [−1.37, −0.03], respectively) from baseline to 12 weeks. Anxiety scores were reduced in both groups (AAC+Ed: −0.72 [−1.15, −0.29] and Control+Ed: −0.52 [−0.96, −0.08]) over 12 weeks (Table 2).

**Table 2:** Baseline and 12-Week Scores and Within- and Between-Group Changes in Psychosocial Measures Assessed by the DASS-21 in the AAC+Ed and Control+Ed Groups

	AAC+Ed		Control+Ed			
Depression, Anxiety, Stress Scales (DASS)	*n*	Median (IQR) or mean (95% CI)	*n*	Median (IQR) or mean (95% CI)	Mean net difference for change^a^	*p*-value^b^
Depression score						
Baseline	121	2 (0–6)	119	2 (0–6)		
12 weeks	115	2 (0–4)	112	2 (0–4)		
Within-group change^c^	115	**−1.00 (−1.65, −0.36)^⁎⁎^**	112	−0.50 (−1.15, 0.15)	−0.50 (−1.42, 0.42)	0.282
Anxiety score						
Baseline	121	2 (0–4)	119	2 (0–4)		
12 weeks	115	0 (0–2)	112	2 (0–2)		
Within-group change^c^	115	**−0.72 (−1.15, −0.29)^⁎⁎^**	112	−0.52 (−0.96, −0.08)*	−0.20 (−0.81, 0.41)	0.520
Stress score						
Baseline	121	4 (2–10)	119	4 (0–8)		
12 weeks	115	4 (0–8)	112	3 (0–8)		
Within-group change^c^	115	−0.69 (−1.37, −0.03)*	112	−0.48 (−1.17, 0.22)	−0.21 (−1.18, 0.76)	0.672

*Note*: Boldface indicates statistical significance (^⁎⁎^*p*<0.01 and *p<0.05).

Asterisks denote statistically significant within-group changes from baseline to 12 weeks, with negative numbers indicating improvement (total scores ranged from 0 to 26 [best to worse]). Within-group changes and mean between-group differences for the change over time were estimated using linear mixed models, with values representing means and 95% CI. Baseline and 12-week data were estimated using descriptive statistics in Stata, with medians (IQR) reported.

a Difference in change between AAC+Ed and Control+Ed groups.

b *p*-values from linear mixed models for the change between groups.

c Baseline-adjusted within-group change from postestimation margins; mean (95% CI). AAC, abdominal aortic calcification; DASS, Depression, Anxiety Stress Scale; Ed, education.

**Table 3:** Baseline and 12-Week Scores and Within- and Between-Group Changes in SF-36 Mental Health Component Summary and Domains in the AAC+Ed and Control+Ed Groups

	AAC+Ed		Control+Ed			
SF-36 domains	*n*	Mean±SD or (95% CI)	*n*	Mean±SD or (95% CI)	Mean net difference for change (95% CI)^a^	*p*-value^b^
Mental health component summary score						
Baseline	120	80.41±16.74	119	81.48±16.48		
12 weeks	115	82.26±15.45	112	81.97±15.88		
Within-group change^c^	115	2.12 (0.03, 4.21)*	112	0.43 (-1.69, 2.55)	1.69 (−1.29, 4.67)	0.266
Role-emotional						
Baseline	121	82.64±33.64	119	86.55±28.23		
12 weeks	115	88.70±26.08	112	87.80±25.70		
Within-group change^c^	115	6.07 (0.92, 11.22)*	112	1.37 (−3.85, 6.58)	4.70 (−2.63, 12.03)	0.209
Energy/fatigue (vitality)						
Baseline	120	66.58±17.48	119	67.90±20.06		
12-weeks	115	67.09±18.98	112	68.17±20.58		
Within-group change^c^	115	0.80 (−1.37, 2.97)	112	−0.09 (−2.29, 2.10)	0.89 (−2.19, 3.98)	0.571
Wellbeing						
Baseline	120	82.37±13.36	119	83.03±13.47		
12-weeks	115	84.03±12.79	112	83.29±13.89		
Within-group change^c^	115	1.77 (0.10, 3.45)*	112	0.50 (−1.19, 2.19)	1.28 (−1.10, 3.66)	0.293
Social functioning						
Baseline	121	88.84±17.96	119	88.45±18.13		
12 weeks	115	89.24±18.50	112	88.62±17.72		
Within-group change^c^	115	0.25 (−2.96, 3.45)	112	−0.04 (−3.28, 3.21)	0.28 (−4.28, 4.84)	0.904

*Note*: Boldface indicates statistical significance (*p*<0.05).

* statistically significant within-group changes (improvements) from baseline to 12 weeks, with mental health component summary score ranging from 0 to 100 (poor to best mental health). Within-group changes and mean between-group differences for the change over time were estimated using linear mixed models, with values representing means and 95% CI. Baseline and 12-week data were estimated using descriptive statistics in Stata, with means±SD reported.

a Difference in change between AAC+Ed and Control+Ed groups.

b *p*-values from linear mixed models for the change between groups.

c Baseline-adjusted within-group change from postestimation margins; mean (95% CI). AAC, abdominal aortic calcification, Ed, education; SF-36, 36-item Short-Form Health Survey.

**Table 4:** Baseline and 12-Week Scores and Within- and Between-Group Changes in Suboptimal Health Status in the AAC+Ed and Control+Ed Groups

	AAC+Ed		Control+Ed			
Suboptimal Health Status domains	*n*	Mean±SD or (95% CI)	*n*	Mean±SD or (95% CI)	Mean net difference for change (95% CI)^a^	*p*-value^b^
Total SHS score						
Baseline	121	38.63±8.44	119	37.87±8.04		
12 weeks	115	37.37±7.71	112	37.30±8.74		
Within-group change^c^	115	**−1.32 (−2.19, −0.45)^⁎⁎^**	112	−0.47 (−1.35, 0.41)	−0.84 (−2.08, 0.39)	0.181
Fatigue						
Baseline	121	14.94±4.22	119	14.87±3.97		
12 weeks	115	14.65±3.97	112	14.52±4.16		
Within-group change^c^	115	−0.29 (−0.79, 0.20)	112	−0.27 (−0.77, 0.23)	−0.02 (−0.72, 0.68)	0.953
Cardiovascular system						
Baseline	121	3.45±0.75	119	3.45±0.81		
12 weeks	115	3.29±0.61	112	3.47±1.00		
Within-group change^c^	115	−0.15 (−0.29, −0.02)*	112	0.02 (−0.11, 0.16)	−0.18 (−0.37, 0.02)	0.071
Digestive system						
Baseline	121	4.05±1.30	119	3.68±1.00		
12 weeks	115	3.90±1.21	112	3.61±0.93		
Within-group change^c^	115	−0.15 (−0.30, −0.01)*	112	−0.08 (−0.23, 0.06)	−0.07 (−0.27, 0.14)	0.510
Immune system						
Baseline	121	4.13±1.28	119	3.97±1.30		
12 weeks	115	4.03±1.27	112	4.04±1.19		
Within-group change^c^	115	−0.12 (−0.32, 0.09)	112	0.08 (−0.14, 0.29)	−0.19 (−0.49, 0.11)	0.210
Mental health						
Baseline	121	12.06±3.26	119	11.89±3.24		
12 weeks	115	11.49±3.14	112	11.66±3.60		
Within-group change^c^	115	**−0.59 (−0.97, −0.21)^⁎⁎^**	112	−0.22 (−0.61, 0.16)	−0.37 (−0.91, 0.17)	0.182
Sleep quality^d^						
Pittsburgh sleep score						
Baseline, mean (IQR)	119	6 (3–10)	116	5 (3–7)		
12 weeks, mean (IQR)	114	6 (3–9)	111	4 (2–7)		
Within-group change^c^	114	−0.11 (−0.53, 0.31)	111	−0.20 (−0.63, 0.23)	0.09 (−0.52, 0.69)	0.774

*Note*: Boldface indicates statistical significance (^⁎⁎^*p*<0.01, *p<0.05).

Asterisks indicate statistically significant within-group changes from baseline to 12 weeks, with negative numbers indicating improvement (SHS score ranges from 0 to 100 [better to poorer suboptimal health]), assessed using the SHSQ-25.^39^ Within-group changes and mean between-group differences for the change over time were estimated using linear mixed models, with values representing means and 95% CI. Baseline and 12-week data were estimated using descriptive statistics in Stata, with mean±SD or median (IQR) reported.

a Difference in change between AAC+Ed versus Control+Ed groups.

b *p*-values from linear mixed models for the change between groups.

c Baseline-adjusted within-group change from postestimation margins; mean (95% CI).

d Sleep quality is not a component of the SHSQ-25. AAC, abdominal aortic calcification; Ed, education; SHS, suboptimal health status; SHSQ-25, 25-item Suboptimal Health Status Questionnaire.

Significant improvements over 12 weeks in mental health component summary score assessed by the SF-36 were observed in AAC+Ed but not in Control+Ed (Table 3). AAC+Ed only also experienced improvements over 12 weeks in role-emotional and well-being; there were no significant within-group differences for vitality or social functioning in either group.

Suboptimal health status improved over 12 weeks in AAC+Ed only (Table 4). Similarly, cardiovascular, digestive, and mental health domain measures from the SHSQ-25 improved significantly over 12 weeks in AAC+Ed but not in Control+Ed. Health status measures for fatigue and immune system as well as sleep quality assessed by the Pittsburgh Sleep Quality Index did not change significantly over time in either group (Table 4).

Findings from sensitivity analyses (Appendix Table 3, available online) indicated an inverse correlation between AAC scores at baseline and changes over 12 weeks in depression scores (rho= −0.230, *p*=0.014) in the AAC+Ed group (i.e., the higher the AAC scores at baseline, the greater the reduction in depression score from baseline to 12 weeks). No correlations were found between baseline AAC scores and changes in anxiety and stress scores. There was a positive association between higher AAC scores and changes (worse) in sleep quality (rho=0.195, *p*=0.039) only in those with evidence of AAC (scores >1). This indicates poorer sleep quality in those with higher AAC scores within the AAC+Ed group. There were no significant differences in changes in any of the outcomes over 12 weeks when comparing those with no evidence of AAC (AAC=0, *n*=50) with those with evidence of any AAC (AAC ≥1, *n*=65) (data not shown).

## DISCUSSION

Findings from this secondary analysis of a 12-week RCT indicate no evidence of adverse or beneficial effects of the provision of AAC imaging results on psychosocial, mental, and suboptimal health measures in older Australian males and females. In particular, providing AAC imaging results with education on CVD risk factors to older males and females did not lead to significant changes in psychosocial measures (depression, anxiety, and stress assessed using DASS-21), mental health (assessed using the SF-36), and suboptimal health status (assessed using the SHSQ-25) compared with education alone.

Despite a statistically significant decrease in depression and stress scores in the AAC+Ed group and decreased anxiety scores in both groups from baseline to 12 weeks, it is important to note that 84%–94% of participants (Table 1) were within the normal reference range^36^ for DASS at baseline (Appendix Table 1, available online). Importantly, the observed changes (<1-point decrease in DASS scales) may not be clinically significant, because a 3.86-point change in depression has been suggested as being clinically meaningful in people with relatively normal depression scores.^44^ Furthermore, the DASS is based on the past week only, and this magnitude of change in the scores could be reflected by changes in 1–2 questions only, with participants remaining in the normal DASS reference range despite these changes, reflecting a normal variation in scores. The decrease in DASS scores may be linked to an increased motivation elicited by the education provided to both groups but could also be related to other reasons, such as life events. The Hawthorne effect could have also played a role.^45^ Participation in clinical trials is a known trigger for changes owing to participants being observed, assessed, or monitored.^45^ As previously reported,^7^ this study was substantially impacted by the COVID-19 pandemic, which may have also been associated with the DASS changes^46^ as the pandemic eased. Overall, the significant decrease in DASS scores over 12 weeks may be due to factors not related to the provision of AAC per se.

Interestingly, participants in the AAC+Ed group reported perceived improvements in their mental health component summary score (SF-36), including its domains (i.e., role-emotional and well-being), and suboptimal health status and its individual domains (i.e., cardiovascular, digestive, and mental health status) over 12 weeks. Suboptimal health status scores focus on predictive, preventive, and personalized health for precision medicine and have been associated with CVD risk factors and may predict CVD-related chronic diseases.^47^

Awareness of being susceptible to a health condition, particularly if the issue may have severe consequences or a poor prognosis, can be perceived as a serious life stressor or threat by some individuals.^21^^,^^48^ The responses to perceived threats vary depending on biological factors; psychosocial aspects; coping mechanisms; cognitive regulation; and other variables, including the amount, severity, and time exposed to stressors.^48^^,^^49^ The presence of a threat, such as the provision of vascular imaging results, can prompt modifications of lifestyle and health behaviors, such as dietary changes and greater adherence to health advice and medications,^13^^,^^50^^,^^51^ which may consequently improve lipid profiles and other health outcomes. However, not all individuals respond in the same way to a threatening situation, with only a small number of studies having assessed psychosocial, mental, and suboptimal health measures after the provision of vascular calcification.^28^ For example, some RCTs reported no significant changes in these measures^24^, ^25^, ^26^^,^^52^ in asymptomatic U.S.-army personnel aged 39–45 years,^24^ asymptomatic sportsmen aged >45 years,^26^ and smokers aged 40–70 years.^52^ The results of this study are also consistent with these findings and the results of a meta-analysis that reported no evidence of beneficial or harmful effects of the provision of vascular imaging results on mental health measures.^28^ However, the within-group perceived improvements in psychosocial, mental, and suboptimal health measures reported by participants suggest that the education provided to them may have prompted some positive changes.

Findings of this study suggest that provision of AAC imaging results along with education aimed at encouraging healthy lifestyle changes did not affect psychosocial, mental, and suboptimal health measures compared with provision of education alone over 12 weeks. These results are encouraging because provision of vascular calcification imaging results to patients has been shown to improve the understanding of CVD risk and may therefore lead to beneficial changes to individuals’ diet and lifestyle, aiming at reducing their risk.^53^ Vascular health screening also provides an opportunity for health professionals to manage CVD risk factors early, aiming at preventing the progression of CVD leading to events.^53^ It can also inform personalized strategies for CVD prevention.^54^

### Limitations

Strengths of this study include the diversity of secondary outcomes assessed at baseline and 12 weeks, including psychosocial, mental, and suboptimal health measures. All questionnaires used were validated and extensively used in Australia and internationally. Investigating the impact of the provision of AAC imaging results on psychosocial, mental, and suboptimal health measures in a randomized controlled setting is a strength per se. However, the results of this study should be interpreted with several limitations considered. Participants may not have clearly understood the AAC results provided to them because they received these through email and discussed them over the phone instead of in person (owing to the COVID-19 restrictions during recruitment). The participants were asked to complete several online questionnaires, which may have been onerous and may have led to inaccurate answers. The study sample size was smaller than the 300 participants planned for recruitment (*n*=227; *n*=115 AAC+Ed and *n*=112 Control+Ed). The significant change in behavior in the AAC+Ed group may have been influenced by the Hawthorne effect, meaning that individuals may have improved or changed over 12 weeks owing to being assessed or monitored as part of an intervention. As previously suggested,^55^ future studies would benefit from having a gap of at least 1 month between enrollment and randomization as well as reassessing participants at the intervention visit, to prevent behavior change prompted by initiating a trial.^55^ The education and advice provided to participants were specific to diet and physical activity behavior changes and did not include stress and mental health–related support. Finally, although the power analysis for the MODEL study was based on changes over 12 weeks in the study’s primary outcomes (objective and subjective measures of consumption of fruit and vegetable intake), a posthoc analysis of the secondary outcomes estimated 99% power to detect a change of 3.86 points in depression (*n*=227, SD=5), which is a clinically meaningful difference reported in people with relatively normal depression scores,^44^ and 80% power to detect a difference ≥5.7 points in mental health component summary score.^56^ Still, these findings should be interpreted with appropriate caution, given that participants received lifestyle advice as a form of support after the provision of vascular calcification results. Participants also had very low scores of depression, anxiety, and stress scales and demonstrated good mental health, as indicated by mental health component summary scores ≥47, comparable to the general Australian population.

## CONCLUSIONS

In conclusion, in this 12-week RCT, provision of AAC results with educational resources did not alter psychosocial, mental, and suboptimal health measures compared with provision of education alone. Further clarification of the psychosocial outcomes after the provision of AAC imaging results is needed and may be achieved by incorporating psychological assessments and support for individuals receiving images of their vascular health in future trials. In addition, longer RCTs with larger and more diverse populations are warranted.

## Appendix Supplementary materials

Image, application 1

## Footnotes

Supplementary material associated with this article can be found, in the online version, at doi:10.1016/j.focus.2026.100507.
