# Supplementary material for: Psychosocial, Mental, and Suboptimal Health Measures Are Not Affected by Provision of Abdominal Aortic Calcification Imaging Results: A 12-Week RCT
Source: AJPM Focus. 2026 May 9;5(6):100507. doi: 10.1016/j.focus.2026.100507 (PMC13634514; doi:10.1016/j.focus.2026.100507)
Supplement: Supplementary file 1 [file mmc1.pdf]

Psychosocial, mental and suboptimal health measures are not affected by provision of abdominal aortic calcification imaging results: a 12-week RCT

American Journal Preventive Medicine (AJPM) Focus

Simone Radavelli-Bagatini[a*], Lauren C. Blekkenhorst[a,b], Catherine P. Bondonno[a,b], Jack Dalla Via[a,c], Marc Sim[a,b], Abadi K. Gebre[a], James Dimmock[d], John T. Schousboe[e,f], Richard J. Woodman[g], Kun Zhu[b,h], Pawel Szulc[i], Ben Jackson[j,k], Wei Wang[l], Markus P. Schlaich[b,m,n,o], Douglas P. Kiel[p,q], Wai H. Lim[a,b,r], Amanda Devine[a], Moira Sim[a], Robin M. Daly[c], Jonathan M. Hodgson[a,b] & Joshua R. Lewis[a,b]

These authors contributed equally: Jonathan M. Hodgson and Joshua R. Lewis.

[a]Nutrition and Health Innovation Research Institute, School of Medical and Health Sciences, Edith Cowan University, Joondalup, WA, Australia.
[b]Medical School, The University of Western Australia, Perth, WA, Australia.
[c]Institute for Physical Activity and Nutrition (IPAN), School of Exercise and Nutrition Sciences, Deakin University, Geelong, VIC, Australia.
[d]Department of Psychology, College of Healthcare Sciences, James Cook University, Townsville, QLD, Australia.
[e]Park Nicollet Clinic and HealthPartners Institute, Minneapolis, MN, USA.
[f]Division of Health Policy and Management, University of Minnesota, Minneapolis, MN, USA.
[g]Flinders Centre for Epidemiology and Biostatistics, Flinders University, Adelaide, SA, Australia.
[h]Department of Endocrinology and Diabetes, Sir Charles Gairdner Hospital, Nedlands, WA, Australia.
[i]INSERM UMR 1033, University of Lyon, Hospices Civils de Lyon, Lyon, France.
[j]The Kids Research Institute Australia, Perth, WA, Australia.
[k]School of Human Sciences (Exercise and Sport Science), University of Western Australia, Perth, WA, Australia.
[l]School of Medical and Health Sciences, Edith Cowan University, Joondalup, WA, Australia
[m]Dobney Hypertension Centre, Medical School, Royal Perth Hospital Research Foundation, Perth, WA, Australia.
[n]Department of Nephrology, Royal Perth Hospital, Perth, WA, Australia.
[o]Department of Cardiology, Royal Perth Hospital, Perth, WA, Australia.
[p]Hinda and Arthur Marcus Institute for Aging Research, Hebrew SeniorLife, Boston, MA, USA.
[q]Department of Medicine, Beth Israel Deaconess Medical Center, Harvard Medical School, Boston, MA, USA.
[r]Department of Renal Medicine, Sir Charles Gairdner Hospital, Perth, WA, Australia.

Supplementary Material

Supplementary Figures

Appendix Figure 1. The MODEL study flow chart

Supplementary Tables

Appendix Table 1. Recommended cut-off scores for Depression, Anxiety, Stress Scale (DASS-21)

Appendix Table 2. Questions comprised in each domain of the Suboptimal Health Status Questionnaire (SHSQ25)

Appendix Table 3. Correlation between baseline AAC24 scores and 12-week change in psychosocial, mental and suboptimal health measures within AAC plus education (AAC+Ed).

Supplementary Figures

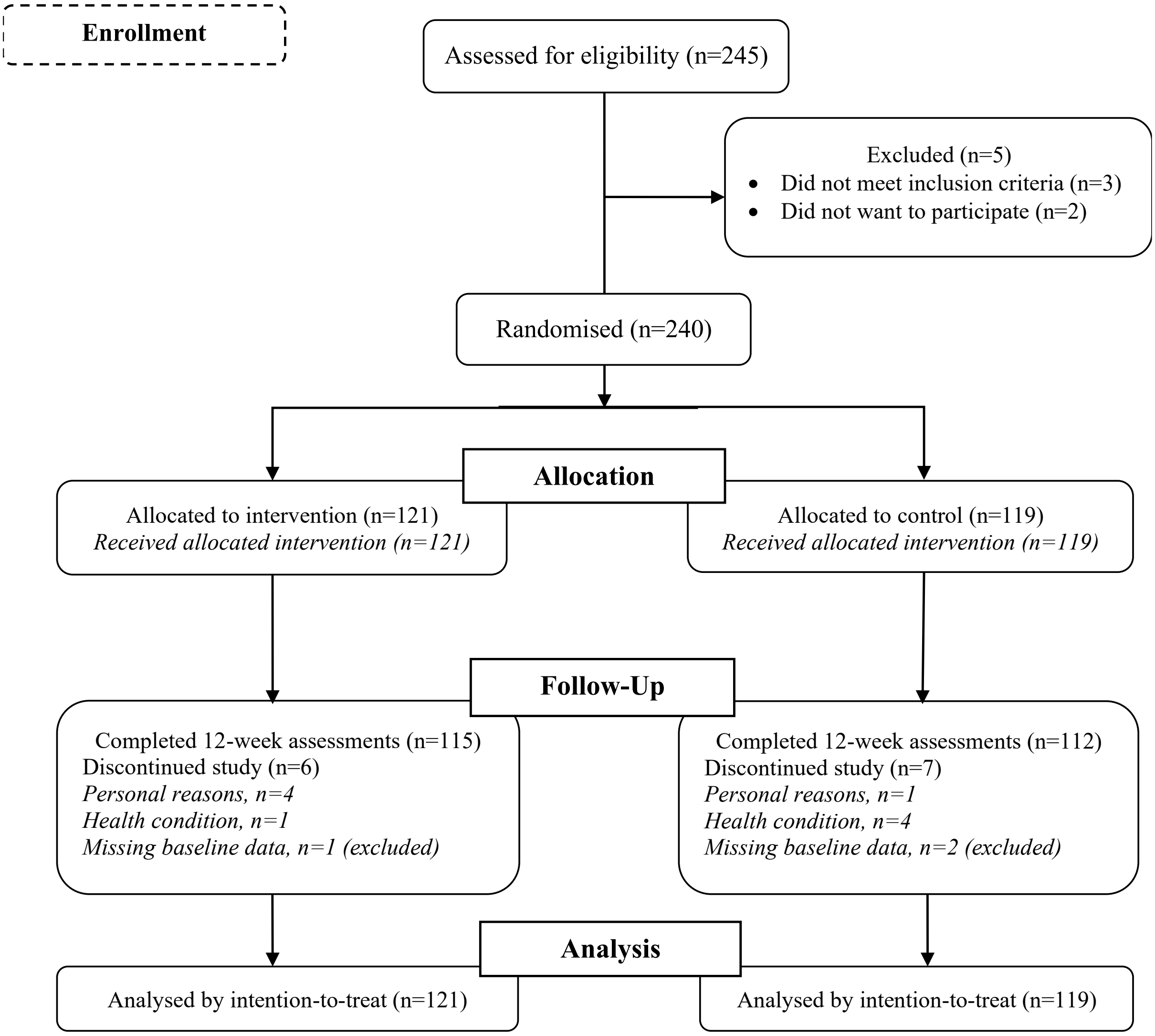

Appendix Figure 1. The MODEL study flow chart

Supplementary Tables

Appendix Table 1. Recommended cut-off scores for Depression, Anxiety, Stress Scale (DASS-21)

DASS-21	Depression	Anxiety	Stress
Normal	0-9	0-7	0-14
Mild	10-13	8-9	15-18
Moderate	14-20	10-14	19-25
Severe	21-27	15-19	26-33
Extremely Severe	≥28	≥20	≥34

The 'mild' category represents scores above the population mean at a level of severity that is lower than what is typically seen in individuals seeking help. Values ≥28, ≥20 and ≥34 indicate elevated risk of depression, anxiety and stress scores, respectively. **Source**: Lovibond PF, Lovibond SH. The structure of negative emotional states: Comparison of the Depression Anxiety Stress Scales (DASS) with the Beck Depression and Anxiety Inventories. Behav Res Ther. 1995;33(3):335-43.

Appendix Table 2. Questions comprised in each domain of the Suboptimal Health Status Questionnaire (SHSQ25)

**Domain 1. Fatigue**
1. In the past 3 months, how often were you exhausted without greatly increasing your physical activity?
2. In the past 3 months, how often did you have fatigue which could not be substantially alleviated by rest?
3. In the past 3 months, how often were you lethargic in your daily life?
4. In the past 3 months, how often did you suffer from headaches?
5. In the past 3 months, how often did you suffer from dizziness?
6. In the past 3 months, how often did your eyes ache or feel tired?
7. In the past 3 months, how often did your muscles or joints feel stiff?
8. In the past 3 months, how often did you have pain in your shoulders / neck / back?
9. In the past 3 months, how often did you have a heavy feeling in your legs when walking?
**Domain 2. Cardiovascular System**
10. In the past 3 months, how often did you feel out of breath while resting?
11. In the past 3 months, how often did you suffer from chest congestion?
12. In the past 3 months, how often were you bothered by heart palpitations?
**Domain 3. Digestive System**
13. In the past 3 months, how often was your appetite poor?
14. In the past 3 months, how often did you suffer from heartburn?
15. In the past 3 months, how often did you suffer from nausea?
**Domain 4. Immune System**
16. In the past 3 months, how often did you have difficulty tolerating hot and cold temperatures?
17. In the past 3 months, how often did you catch a cold?
18. In the past 3 months, how often did you suffer from a sore throat?
**Domain 5. Mental Health**
19. In the past 3 months, how often did you have difficulty falling asleep?
20. In the past 3 months, how often were you troubled by waking up during the night?
21. In the past 3 months, how often did you have trouble with your short-term memory?
22. In the past 3 months, how often did you have difficulty responding quickly?
23. In the past 3 months, how often did you have difficulty concentrating?
24. In the past 3 months, how often were you distracted for no reason?
25. In the past 3 months, how often did you feel nervous or jittery?

Response options. Never or almost never, occasionally, often, very often, always. **Source**: Buysse DJ, Reynolds III CF, Monk TH, Berman SR, Kupfer DJ. The Pittsburgh Sleep Quality Index: a new instrument for psychiatric practice and research. Psychiatry Res. 1989;28(2):193-213.

Appendix Table 3. Correlation between baseline AAC24 scores and 12-week change in psychosocial, mental and suboptimal health measures within AAC plus education (AAC+Ed).

	AAC score >1	
**Changes over 12 weeks**	**ρ**	**p-value**
**Psychosocial measures assessed by DASS-21**		
Depression score	**-0.230**	**0.014**
Anxiety score	-0.003	0.740
Stress score	0.042	0.660
**Mental health assessed by SF-36**		
Total mental health component summary score	-0.071	0.452
Role-emotional	0.032	0.737
Energy/fatigue (vitality)	-0.037	0.695
Wellbeing	-0.034	0.718
Social functioning	-0.104	0.274
**Suboptimal health status (SHS) assessed by SHSQ-25**		
Total SHS score	0.160	0.090
Fatigue	0.138	0.144
Cardiovascular system	-0.136	0.152
Digestive system	0.140	0.140
Immune system	-0.016	0.864
Mental health	0.133	0.161
**Sleep quality**		
Pittsburgh Sleep Score	**0.195**	**0.039**

Rho (ρ) is from Spearman's correlation. Bold indicates a significant correlation between higher baseline AAC score and greater change in outcome measures over 12 weeks.
Abbreviations: AAC, abdominal aortic calcification; DASS, Depression, anxiety stress scale; Ed, education; SF-36, Short-Form Health Survey (SF-36) questionnaire, SHSQ, Suboptimal Health Status Questionnaire.
